# Development of a humanized mouse model to analyze antibodies specific for human leukocyte antigen (HLA)

**DOI:** 10.1371/journal.pone.0236614

**Published:** 2021-02-05

**Authors:** Senichiro Yanagawa, Hiroyuki Tahara, Takayuki Shirouzu, Shintaro Kawai, Yuka Tanaka, Kentaro Ide, Shuji Akimoto, Hideki Ohdan

**Affiliations:** 1 Department of Gastroenterological and Transplant Surgery, Graduate School of Biomedical and Health Sciences, Hiroshima University, Hiroshima, Japan; 2 Molecular Diagnostics Division, Wakunaga Pharmaceutical Co., Ltd., Osaka, Japan; University of Kentucky, UNITED STATES

## Abstract

In organ transplantation, human leukocyte antigen (HLA)-mismatch grafts not only induce the activation of cellular mediated immune response but also the development of chronic antibody-mediated rejection due to the donor-specific anti-HLA antibody (DSA) produced by B cells and plasma cells interacting with the graft endothelium. Significant improvement in long-term survival after transplantation can be expected if antibody-mediated rejection due to the DSA can be overcome. However, the mechanism of producing or controlling the DSA remains to be elucidated. In recent decades, “humanized” mouse models have been widely used for the basic research of human immune systems, but a humanized mouse model to analyze the mechanism of DSA production has not been established yet. Thus, we aimed to create a humanized mouse using a severe immunodeficiency mouse (NSG mouse) administered with human peripheral blood mononuclear cells (PBMCs). Initially, we detected a very low level of human total-IgG and no anti-HLA antibodies (Abs) in these mice. In our next attempt, we mixed PBMCs of various HLA antigenic combinations with or without regulatory T cells and preconditioned them by culturing on feeder cells stably transfected with human CD40 ligand (h-CD40L) alone or with h-CD40L and human B cell activating factor (h-BAFF). They were subsequently co-cultured with the corresponding irradiated stimulator PBMCs, and all cells were administered into naïve NSG mice. Although all three humanized models had sufficient human total-IgG and anti-HLA antibody production, allospecific anti-HLA Ab production was prominently suppressed whereas non-specific anti-HLA Abs were sufficiently detected. Therefore, this novel humanized mouse model might be useful for analyzing the mechanism of anti-allogeneic human B cell tolerance induction.

## Introduction

Human leukocyte antigen (HLA) is distributed in nearly all cells and body fluids and functions as a histocompatibility antigen (an important molecule related to human immunity). In organ transplantation, HLA conformity is important because different forms of HLA are recognized as foreign objects which are subject to attack from the immune system; additionally, HLA-mismatched grafts induce the activation of cellular mediated immune response, leading to graft rejection in the absence of immunosuppressive therapy [1–4]. Therefore, appropriate selection of donors is required before transplantation. However, HLA is rich in polymorphisms, and HLA between recipients and donors differ in many cases. Despite recent advances in immunosuppression and antibody (Ab) screening prior to transplantation, chronic antibody-mediated allograft rejection due to donor-specific anti-HLA antibodies (DSA) influence transplantation outcomes; however, the immunological mechanism of antibody-mediated rejection due to DSA is unclear [1, 2]. Elucidation of the process of antibody-mediated rejection is imperative for developing new immune therapies to improve long term prognosis of organ transplants [5]. To develop a therapy that effectively regulates DSA-secreting cells, it is necessary to determine the mechanism underlying DSA production in human immunocompetent cells.

In mouse models of heart transplantation from BALB/c to C57BL/6, the spleen and bone marrow were found to be the major sources of DSA-secreting cells [6]. However, the detection of DSA-secreting cells and mechanism of DSA production in human immune cells *in vivo* have not been widely examined because this analysis in the human clinical setting is difficult. Successful immunotherapies produced in animal models and transplanted into clinical cases have shown limited success because of the many species-specific differences between mouse and human immune responses.

In recent decades, immunodeficient mice used for engraftment with the functional human immune system have been developed, known as “humanized mouse models” [7–11]. These models contain various types of human cells and tissues engrafted in immunodeficient mice and are extremely useful for basic research for studies of the human immune system; however, there is no established humanized mouse model that specifically analyzes the mechanism of human DSA production and the antibody (Ab)-secreting human B cells, especially one that uses human peripheral blood mononuclear cells (PBMCs).

To detect human DSA-secreting cells and the DSA in an *in vivo* model, we attempted to establish of an anti-HLA Ab-producing humanized mouse model by reconstructing human immunocompetent cells. Responder PBMCs preselected for HLA antigens were cultured with or depleted of regulatory T cells on feeder cells expressing human CD40L alone or both CD40L and BAFF (B cell activating factor). The responder PBMCs were co-cultured with irradiated stimulator PBMCs before administration into naïve NSG mice in order to facilitate anti-HLA Ab production.

## Materials and methods

### Ethics statement

This study was performed in strict accordance with the Guide for the Care and Use of Laboratory Animals, and the experimental protocol was approved by the Ethics Review Committee for Animal Experimentation of the Graduate School of Biomedical Sciences, Hiroshima University (Permit Number: A17-64-2). All animal experiments were performed according to the guidelines established by the US National Institutes of Health (1996). This work was carried out, in part, at the Research Facilities for Laboratory Animal Science, Natural Science Center for Basic Research and Development, Hiroshima University.

### Mice

NOD.Cg-Prkdc^scid^IL2rg^tm1Wjl^/SzJ (NSG) mice were purchased from Japan Charles River (Yokohama, Japan). NSG mice carry two mutations in the NOD/ShiLtJ genetic background. The severe combined immune deficiency (*scid*) mutation is in the deoxyribonucleic acid (DNA) repair complex gene *Prkdc* and renders the mice B and T cell deficient. The *IL2rg*^*null*^ mutation prevents cytokine signaling through multiple receptors, leading to a deficiency in functional natural killer (NK) cells. Such severe immunodeficient mice can be expected to improve the engraftment effect of human immunocompetent cells.

NSG mice were maintained and bred in a specific pathogen-free facility in micro-isolator cages at the Natural Science Center for Basic Research and Development Hiroshima University. Daily animal welfare was carefully ensured by experienced operators, and all efforts were made to minimize the suffering of the animals for the duration of their lives.

### Preparation of the human PBMCs

Peripheral blood samples from human recipients or living donors of liver and/or kidney transplants at Hiroshima University Hospital were collected into separate heparinized tubes and centrifuged using phosphate-buffered saline (PBS) (Cat.#05913, Nissui, Tokyo, Japan) and Lympholyte (Cat.#CL5026, Cedarlane Laboratories, Burlington, ON, Canada) at 1500 rpm for 30 min. PBMCs were collected and washed with PBS for the experiments.

### Combinations of HLA alleles in responder and stimulator cells

PBMCs with different combinations of HLA class I molecules were prepared (Table 1). Flow cytometry (FCM) analyses were performed to distinguish the cells based on differences in HLA-A2 or A24. All responders and stimulators were typed for HLA-A, -B, and -C at the HLA Foundation Laboratory, Kyoto, Japan or using a commercially available DNA typing reagent, WAKFlow^R^ HLA DNA Typing (Cat.#4N705,4N805,4P305, Wakunaga Pharmaceutical Co., Ltd., Osaka, Japan).

**Table 1:** Combination HLA of responder and stimulator PBMCs.

Combination 1	Combination 2	Combination 3
Responder HLA	Responder HLA	Responder HLA
A*24:02, A*33:03	A*02:01, A*02:06	A*02:01, -
B*15:18, B*52:01	B*51:01, -	B*13:01, B*40:01
C*01:02, C*12:02	C*08:01, C*14:02	C*03:04, C*15:02
Stimulator HLA	Stimulator HLA	Stimulator HLA
A*02:07, A*26:01	A*24:02, A*33:03	A*24:02, -
B*40:02, B*51:01	B*15:18, B*52:01	B*51:01, B*54:01
C*03:04, C*14:02	C*01:02, C*12:02	C*01:02, C*14:02

### Generating a humanized mouse model simply injected with human PBMCs

As an initial test, responder and stimulator PBMCs with HLA combination 1 (Table 1) were administered to severe immunodeficient naïve NSG mice to produce anti-HLA Abs. Both these PBMCs were not preconditioned in an *in vitro* culture before administration to the naïve NSG mice. Responder PBMCs (10 × 10^6^) were intravenously injected into the mice (Mouse # 1–1 and 1–2), then one day later, they were intraperitoneally challenged with 30Gy gamma-irradiated stimulator PBMCs (10 × 10^6^).

### Transfection of human B cell activating factor (h-BAFF) gene to feeder cell line expressing human CD40 ligand (h-CD40L)

Dr. G. J. Freeman kindly provided the basic feeder cell line, NIH 3T3 h-CD40L, which stably expresses human CD40L [12]. Originally, the NIH 3T3 cells were stably transfected via electroporation with Fsp I linearized h-CD40L plasmid and Pvu I linearized pSP65- Neomycin resistance gene (Neo) plasmid. The transfectants were selected by G418 because resistance to G418 was conferred by the Neo from Tn5 encoding an aminoglycoside 3’-phosphotransferase II.

We further transfected the NIH 3T3 h-CD40L cell line with human B-cell activating factor (h-BAFF). These feeder cells were seeded in 96-well plates in Dulbecco’s Modified Eagle Medium (DMEM) (Cat.#10566–016, ThermoFisher Scientific, Waltham, MA, USA) at 1.5×10^4^ cells/well, and incubated with 5% CO2 at 37°C for 24 h before transfection with h-BAFF. For each well, h-BAFF complementary DNA cloned in the pCMV3-untagged expression vector (0.1 μg) (Cat.#HG10056-UT, Sino Biological Inc, Beijing, China) was diluted in DMEM to a total volume of 25 μL and held at room temperature for 5 min. Meanwhile, 0.75 μL Sinofection transfection reagent (Cat.#STF02, Sino Biological Inc) was diluted in DMEM to total volume of 25 μL and incubated at room temperature for 5 min. The two solutions were then mixed and further incubated at room temperature for 20 min. The supernatant of feeder cells expressing h-CD40L was removed, and the 50 μL mixture of DNA and Sinofection was added to each well and incubated with 5% CO2 at 37°C for 6 h. The supernatant was removed and medium consisting of DMEM/F12 (Dulbecco’s Modified Eagle Medium/Nutriment Mixture F-12) supplemented with GlutaMAX^TM^ (Cat.#10565–018, ThermoFisher Scientific), 50 mg/mL G418 (Cat.#09380–86, Nacalai, Kyoto, Japan), 1M HEPES (Cat.#15630–080, Thermo Fisher Scientific), 10% fetal bovine serum (FBS) (Cat.#04-007-1A, Biological Industries Ltd., Beit Haemek, Israel) was added, and the cells were incubated with 5% CO2 at 37°C for 72 h. After incubation, selection for h-BAFF expressing cells was performed with 800 μg/mL hygromycin B (Cat.#09287–84, Nacalai). After selection, the feeder cells were incubated with medium consisting of DMEM/F12, GlutaMAX^TM^, G418, 1M HEPES, 10% FBS (as above) with 5% CO2 at 37°C for 72 h. The same process of incubation and selection was repeated three times. This process created the new feeder cells expressing h-CD40L and h-BAFF. In FCM analysis, the rate of h-BAFF expression was over 90% (S1 Fig). Both the h-CD40L and h-CD40L/h-BAFF feeder cell lines were used subsequently and were gamma-irradiated (120 Gy) before culturing.

### Depletion of regulatory T cells (T-regs) from the responder PBMCs

Regulatory T cells (T-regs) defined as CD4^+^CD25^+^T cells were depleted from the responder PBMCs using the Easy Sep^TM^ Human CD4^+^CD25^+^ T Cell Isolation Kit (Cat. #18062, StemCell Technologies, Vancouver, Canada), according to the manufacturer’s protocol. In detail, CD4^+^T cells and other cells (depleted CD4^+^T cells) were separated from responder PBMCs by Easy Sep^TM^ Human CD4^+^T cell Enrichment Cocktail and Easy Sep^TM^ D Magnetic Particles and Easy Sep^TM^ Blocking solution. CD4^+^T cells were separated into CD4^+^CD25^+^T cells and CD4^+^CD25^-^T cells by Easy Sep^TM^ Human CD25 positive selection Cocktail and Easy Sep^TM^ Magnetic Nanoparticles. The other cells (depleted CD4^+^T cells) and CD4^+^CD25^-^T cells were mixed. In this way, T-regs as CD4^+^CD25^+^T cells were depleted from Responder PBMCs. We confirmed the removal of about 90% of T-regs by FCM (S2 Fig).

### Generating a humanized mouse model producing anti-HLA Abs

We constructed a second humanized mouse model in which antibody-producing B cells were pre-activated *in vitro* and then transferred to naïve NSG mice. To activate the Ab-producing B cell using the signaling of human CD40-CD40 ligand, two different HLA types of PBMCs (fresh non-irradiated responder and irradiated stimulator PBMCs) were co-cultured with feeder cells expressing h-CD40L for three days with all HLA combinations (Table 1). After co-culture in a humidified atmosphere at 37°C with 5% CO_2_ for three days, cultured PBMCs and feeder cells were collected (20 × 10^6^) and administered to naïve NSG mice by intrasplenic infusion (day 0). On day 1, gamma-irradiated (30Gy) stimulator PBMCs (10 × 10^6^) were challenged to the mice. Prior to intrasplenic infusion, mice were anesthetized by intraperitoneal injection of xylazine (5 mg/kg body weight) and ketamine (100 mg/kg body weight). Based on this procedure, we created three protocols (*in vitro* combinations detailed below) in an attempt to generate the humanized model (S3 Fig).

All experiments were conducted using feeder cells not exceeding 60–70% confluence to avoid pericellular hypoxia induced by over confluency. The cells were collected and counted by the trypan blue dye exclusion method.

The first protocol: Two different HLA types of human PBMCs (fresh non-irradiated responder PBMCs 5 × 10^6^/well and irradiated stimulator PBMCs 5 × 10^6^/well) were co-cultured with feeder cells expressing h-CD40L (0.5 × 10^6^/well) for three days in RPMI-1640 medium (Cat.#11875–093, Thermo Fisher Scientific) supplemented with 10% FBS, 2-mercaptoethanol (Cat.#21438–82, Nacalai), and 1M HEPES in a 6-well plate (total volume 8 mL/well). Mice # 1-1-1 and 1-1-2 received cells with HLA combination 1, # 1-2-1 and 1-2-2 received combination 2, # 1-3-1 and 1-3-2 received combination 3.

The second protocol: Two different HLA types of human PBMCs (fresh non-irradiated responder PBMCs 5 × 10^6^/well and irradiated stimulator PBMCs 5 × 10^6^/well) were co-cultured with feeder cells expressing both h-CD40L and h-BAFF (0.5 × 10^6^/well) for three days using the same condition. Mice # 2-1-1 and 2-1-2 received combination 1, # 2-2-1 and 2-2-2 received combination 2, # 2-3-1 and 2-3-2 received combination 3.

The third protocol: Two different HLA types of human PBMCs (fresh non-irradiated T-regs-depleted responder PBMCs 5 × 10^6^/well and irradiated stimulator PBMCs 5 × 10^6^/well) were co-cultured with feeder cells expressing h-CD40L (0.5 × 10^6^/well) for three days under the same conditions. Mice # 3-1-1 and 3-1-2 received combination 1, # 3-2-1 and 3-2-2 received combination 2, # 3-3-1 and 3-3-2 received combination 3.

In all 9 experimental mice (#1-1-1 to #3-3-2), the proportion of h-CD45 positive cells was confirmed by FCM in the cells collected after the *in vitro* culture. The proportion of h-CD45 positive cells was 88.4 ± 2.5% (mean ± standard error, range 71.9–95.7).

Table 2 summarizes the three protocols and *in vitro* co-culture conditions to generate the three different types of humanized mouse model, and the identification number of each experimental mouse.

**Table 2:** Humanized mouse protocols and pre-culture conditions.

Protocol	Feeder expressing h-CD40L	Feeder expressing h-BAFF	Depletion of T-regs	Mouse number
**First**	+	-	-	# 1-1-1, 1-1-2, 1-2-1, 1-2-2, 1-3-1, 1-3-2
**Second**	+	+	-	# 2-1-1, 2-1-2, 2-2-1, 2-2-2, 2-3-1, 2-3-2
**Third**	+	-	+	# 3-1-1, 3-1-2, 3-2-1, 3-2-2, 3-3-1, 3-3-2

### FCM analysis

Cell suspensions were prepared from peripheral blood of humanized mice at 2 weeks post-injection (day 14) and the chimera state was confirmed. These samples were stained with fluorescein isothiocyanate (FITC)-conjugated anti-HLA-A2 and anti-human CD257 (BAFF); phycoerythrin (PE)-conjugated anti-human CD25, CD45; and allophycocyanin (APC)-conjugated anti-human CD3, CD4, CD14, CD19 Abs purchased from eBioscience (San Diego, CA, USA), Medical & Biological Laboratories Co., Ltd. (Nagoya, Japan) and BD Pharmingen (San Diego, CA, USA). Flow cytometry was carried out on a FACS Canto flow cytometer (Becton Dickinson, Mountain View, CA) using FlowJo^TM^_v10.6.2 software (Tree Star Inc., Ashland, OR, USA). Dead cells, identified by light scatter and propidium iodide staining, were excluded from the analysis.

### Measurement of human total-IgG and anti-HLA antibody titers

To measure human total-IgG in the sera of humanized mice at pre-injection and 1–5 weeks (day 7 to 35) post-injection, we used the BD^TM^ Cytometric Bead Array (CBA) Human Total IgG Flex Set (Cat.#558679) and FCAP Array^TM^ v3.0 Software (both BD Biosciences, Franklin Lakes, NJ, USA). Anti-HLA Abs are reported as the mean fluorescent intensity (MFI) in the sera with highest levels of human total-IgG; MFI 100 or more was judged to be anti-HLA antibody titer-positive measured by the Luminex assay using WAKFlow^R^ class Ⅰ antibody specificity identification reagent (Cat.#4KV02, Wakunaga Pharmaceutical Co., Ltd., Japan). We set MFI 100 or more to indicate positively, because MFI was less than 20 in all cases in the sera of naïve NSG mice prior to human PBMCs transfer. In addition, the MFI of the negative control serum used in this Luminex assay was less than 100 throughout all experiments.

### Statistical analysis

We compared the anti-HLA antibody-producing ability between the allospecific and non-specific anti-HLA Abs using the Welch’s *t* test. *P*-values of 0.05 or less were considered statistically significant. All statistical analyses were performed with software ‘EZR’ [13]

## Results

### Very low level of human total-IgG and no anti-HLA Abs were detectable in the humanized mouse model simply injected with human PBMCs

**Fig 1:**
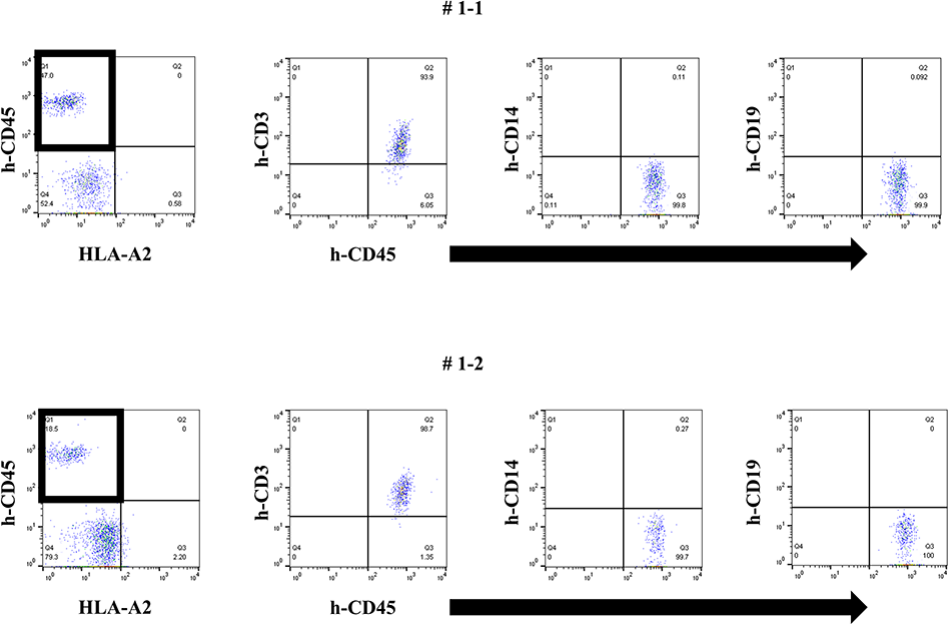
Two types of PBMCs with different HLA types (combination 1) were administered to naïve NSG mice.

**Fig 2:**
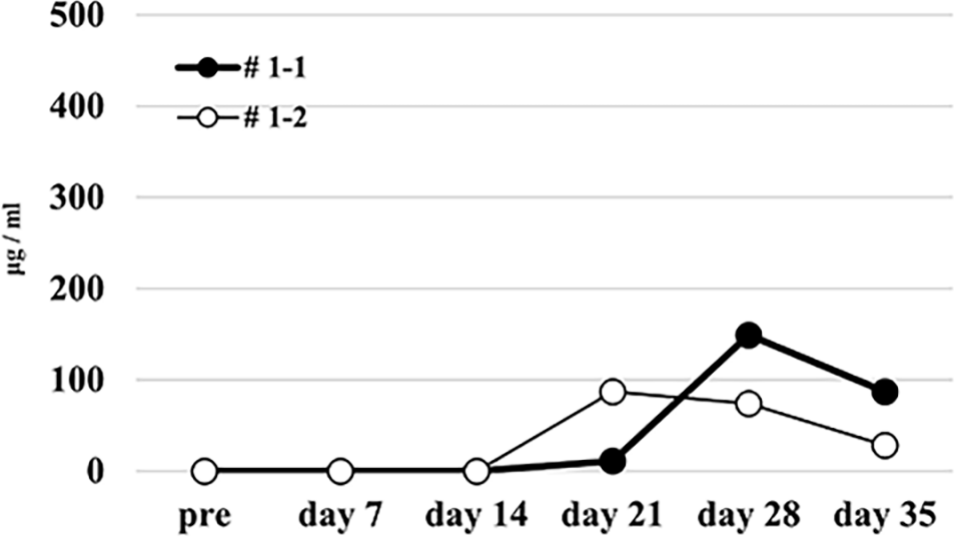
Human total-IgG production in the humanized mouse model simply injected with human PBMCs.

**Fig 3:**
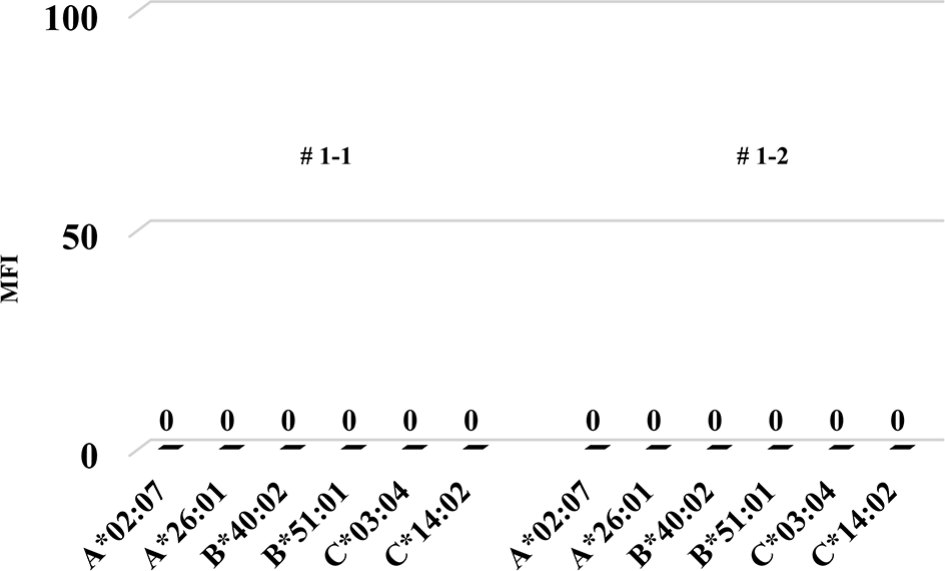
The MFI of anti-HLA Abs in the humanized mouse model simply injected with human PBMCs.

Firstly, we tried to develop a simple humanized mouse model with two different types of PBMC injection (Mouse # 1–1 and 1–2). Because the stimulator PBMCs had been irradiated before intraperitoneal challenging, only the responder PBMCs were detected in the peripheral blood of these mice on day 14 after injection. Since only the stimulator PBMCs had HLA-A2, as shown in Fig 1, h-CD45^+^cells engrafted in these mice were only responder-derived HLA-A2-negative cells. Most of these cells were human CD3-positive cells, but only a few human CD14- and CD19-positive cells were engrafted (Fig 1). In addition, we detected very low level of human total-IgG and no anti-HLA Abs in these mice (Figs 2 and 3).

In FCM on day 14, only responder cells were detected in the peripheral blood of humanized mice and most h-CD45^+^ cells were h-CD3^+^. The cells from the responder are surrounded by square. This chimera status was common in each mouse.

Very low level of human total-IgG was measured in each mouse (Mouse # 1–1 and 1–2).

Anti-HLA antibodies were never produced in each mouse (Mouse # 1–1 and 1–2).

### Human total-IgG and anti-HLA Abs are sharply increased in the first protocol humanized mouse model, but no allospecific anti-HLA Abs are detected

**Fig 4:**
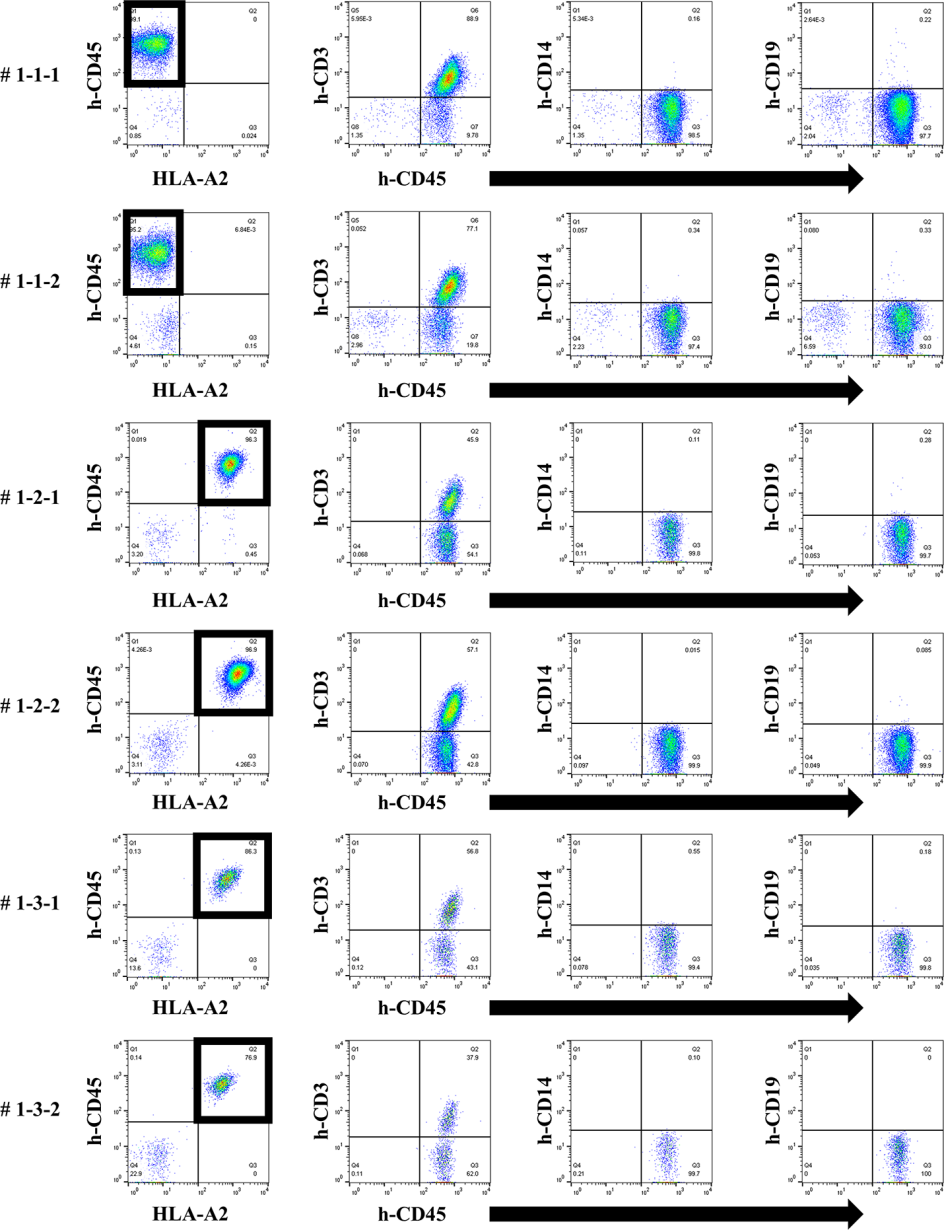
Chimeric status in all humanized mice under the first protocol.

We next attempted to produce a humanized mouse with injection of preconditioned PBMCs. On day 14, only the responder cells were detected by FCM in the peripheral blood of humanized mice treated with the first protocol, and most h-CD45^+^ cells were h-CD3^+^ for all PBMC HLA combinations used (Fig 4). No cells derived from the stimulator PBMCs were detected because of the effect of gamma-irradiation before the co-culture. This chimeric state was similar in all HLA combinations.

**Fig 5:**
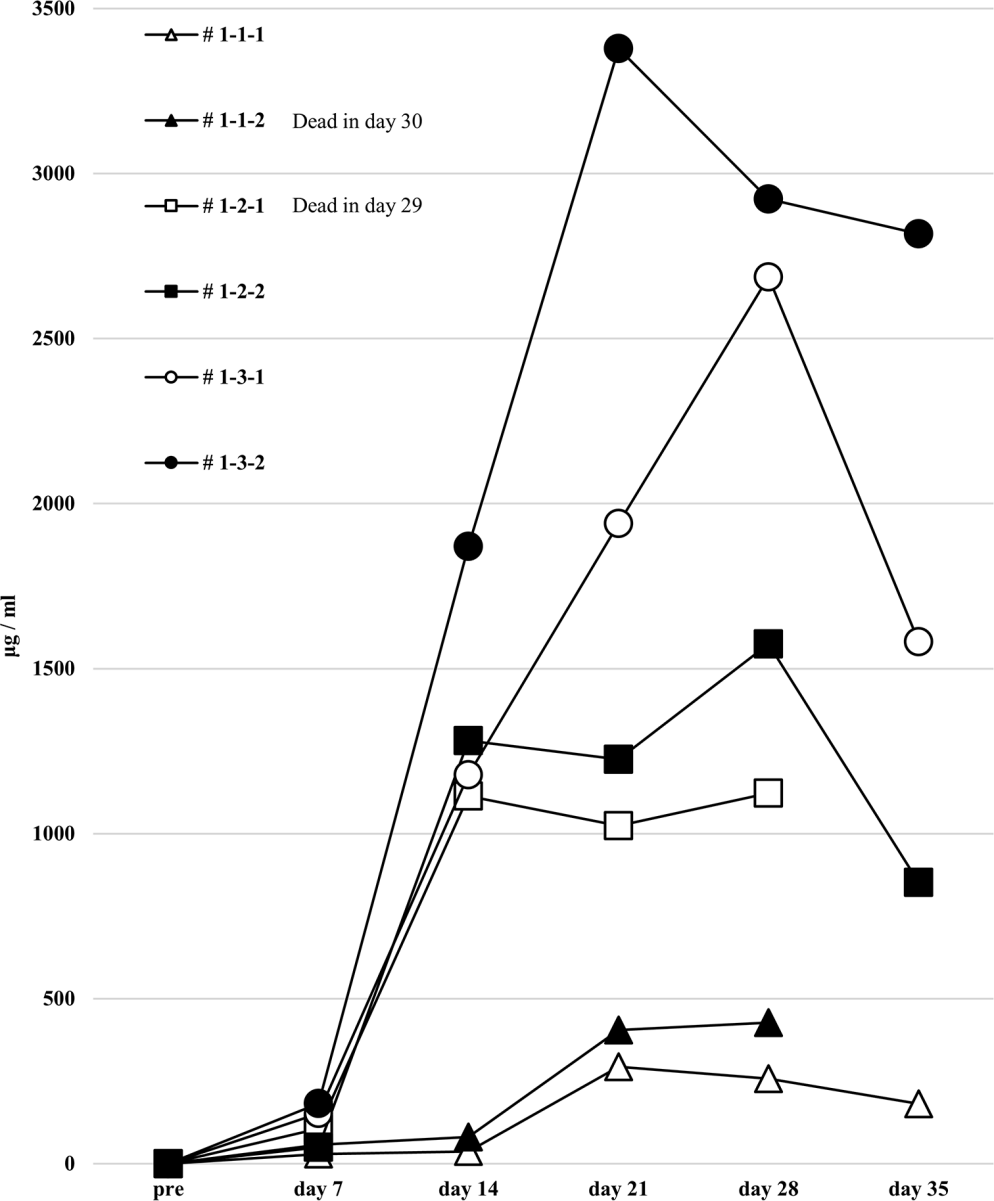
Human total-IgG production in all humanized mice under the first protocol.

**Fig 6:**
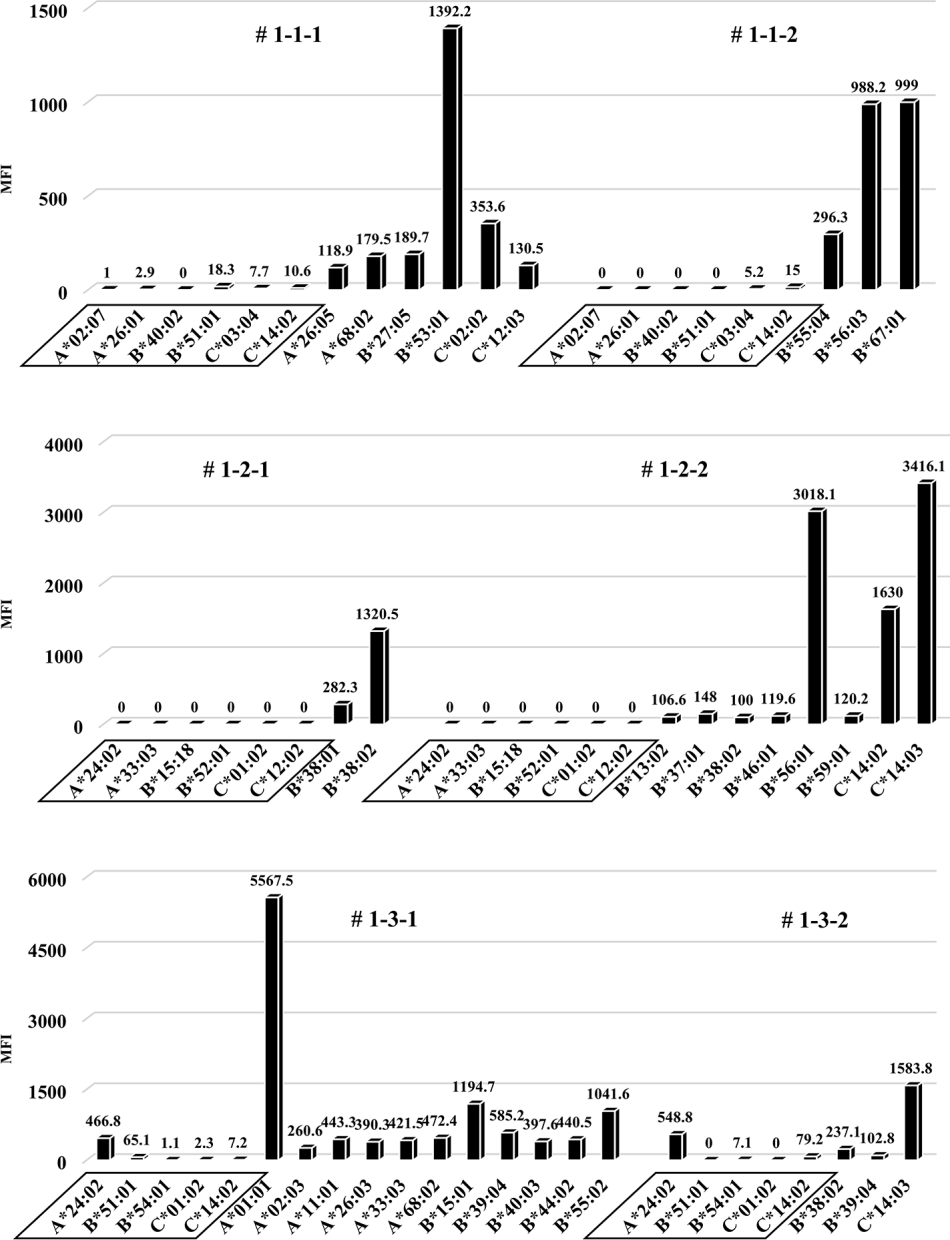
Anti-HLA Abs production in all humanized mice under the first protocol.

In the humanized mouse sera from the first protocol, human total-IgG was increased in NSG mice after administration of the PBMCs (Fig 5). When measuring anti-HLA Ab titers in the sera at the time point of the highest levels of human total-IgG, clearly increased anti-HLA Ab titers were present, but almost all were alloantigen non-specific anti-HLA Abs (Fig 6). With each mouse that received HLA combination 3, only allospecific anti-HLA Ab (A*24:02) was slightly present in the serum, but other allospecific anti-HLA Abs (B*51:01, B*54:01, C*01:02, C*14:02) were not produced. No correlation was observed in the titers between human total-IgG and anti-HLA Abs. Moreover, the abundance ratio of peripheral blood B cells in these humanized mice did not show a clear correlation with the values of human total-IgG or anti-HLA Abs. Further, the mice having higher anti-HLA Ab titers did not necessarily show proportionally higher human total-IgG values.

In FCM on day 14, only responder cells were detected in the peripheral blood of humanized mice under the first protocol. The cells from the responder PBMCs are surrounded by a square. This chimera status was common for all HLA combinations (# 1-1-1, 1-1-2, 1-2-1, 1-2-2, 1-3-1, 1-3-2). The majority of human CD45^+^ cells were human CD3^+^ T cells and human CD19^+^ B cells were found in all but a few of the mice.

Humanized mouse sera were collected before intrasplenic PBMC infusion into naïve NSG mice and at 1–5 weeks (day 7 to 35) after infusion. Human total-IgG was sufficiently elevated in all the humanized mice. Mouse # 1-1-2 was dead at day 30 and # 1-2-1 was dead at day 29.

Quantification of anti-HLA Abs produced in all of the humanized mice under the first protocol are shown. The stimulator HLA allele names (i.e., allospecific anti-HLA Abs) are enclosed in a square. All humanized mice produced various allelic non-specific anti-HLA Abs. However, alloantigen specific anti-HLA Ab (A*24:02) was slightly produced only by mouse # 1-3-1 and # 1-3-2.

### No increase in human total-IgG and whole anti-HLA Abs, and no allospecific anti-HLA Abs produced in the second protocol of humanized mouse model

**Fig 7:**
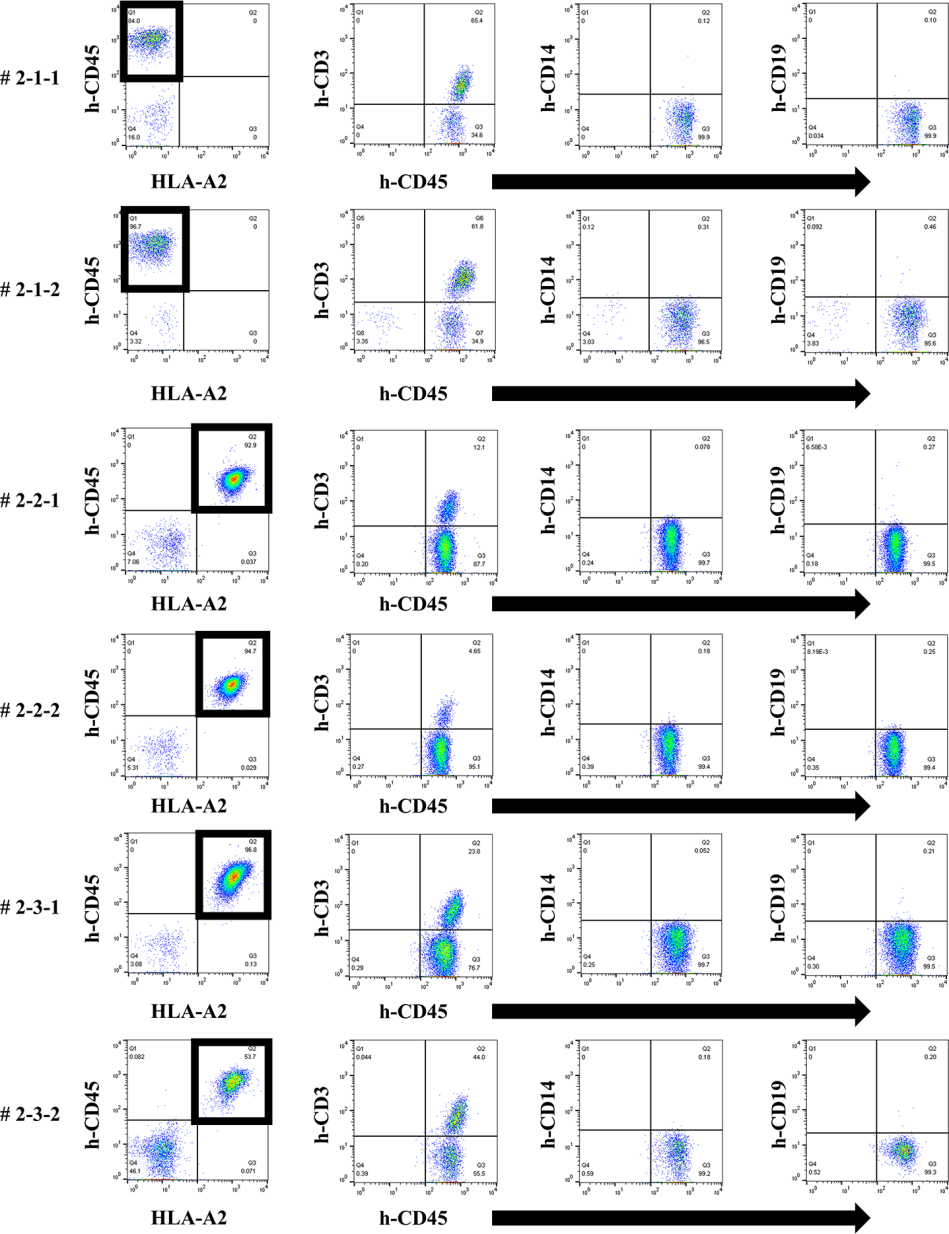
Chimeric status in all humanized mice under the second protocol.

**Fig 8:**
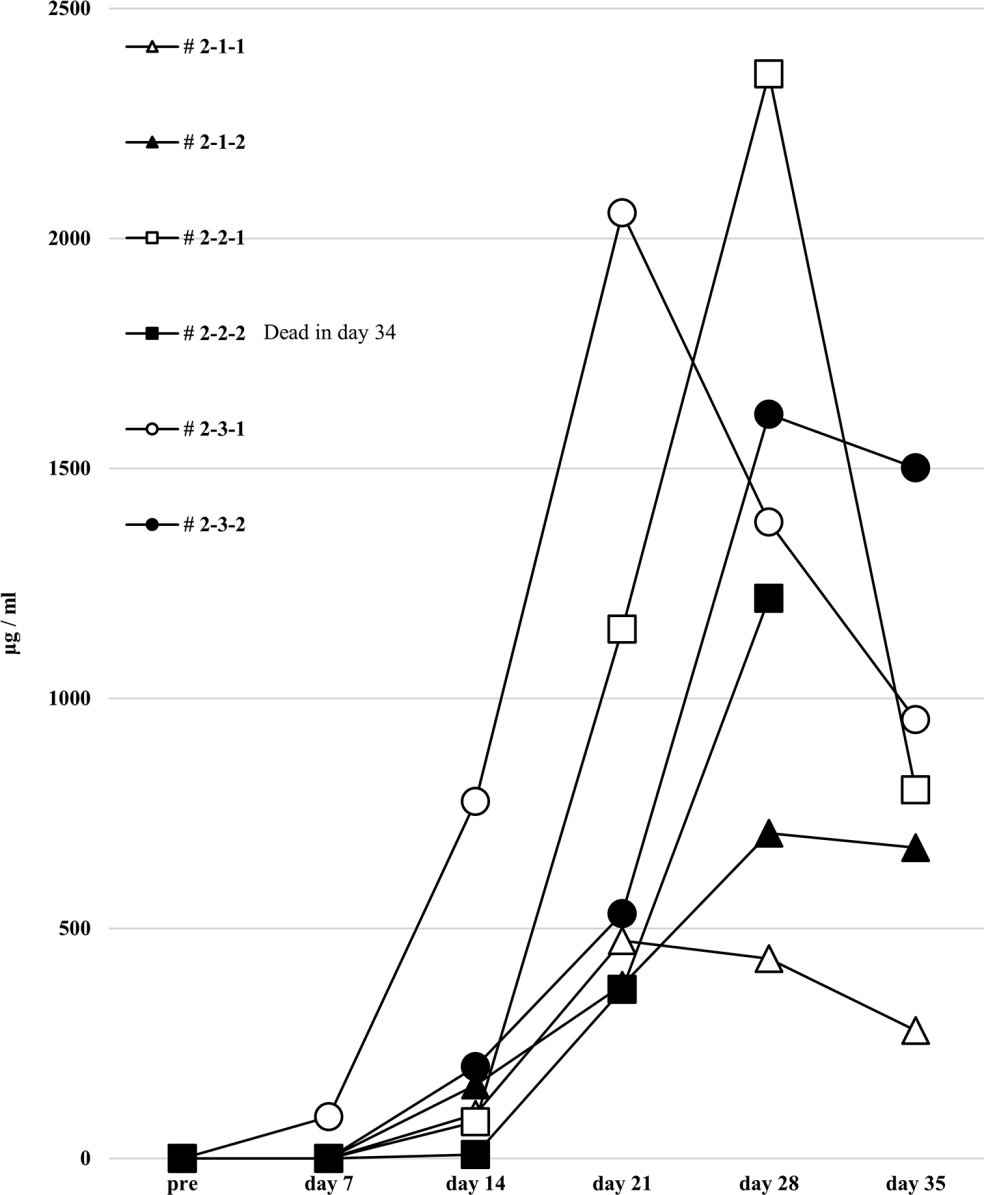
Human total-IgG production in all humanized mice under the second protocol.

**Fig 9:**
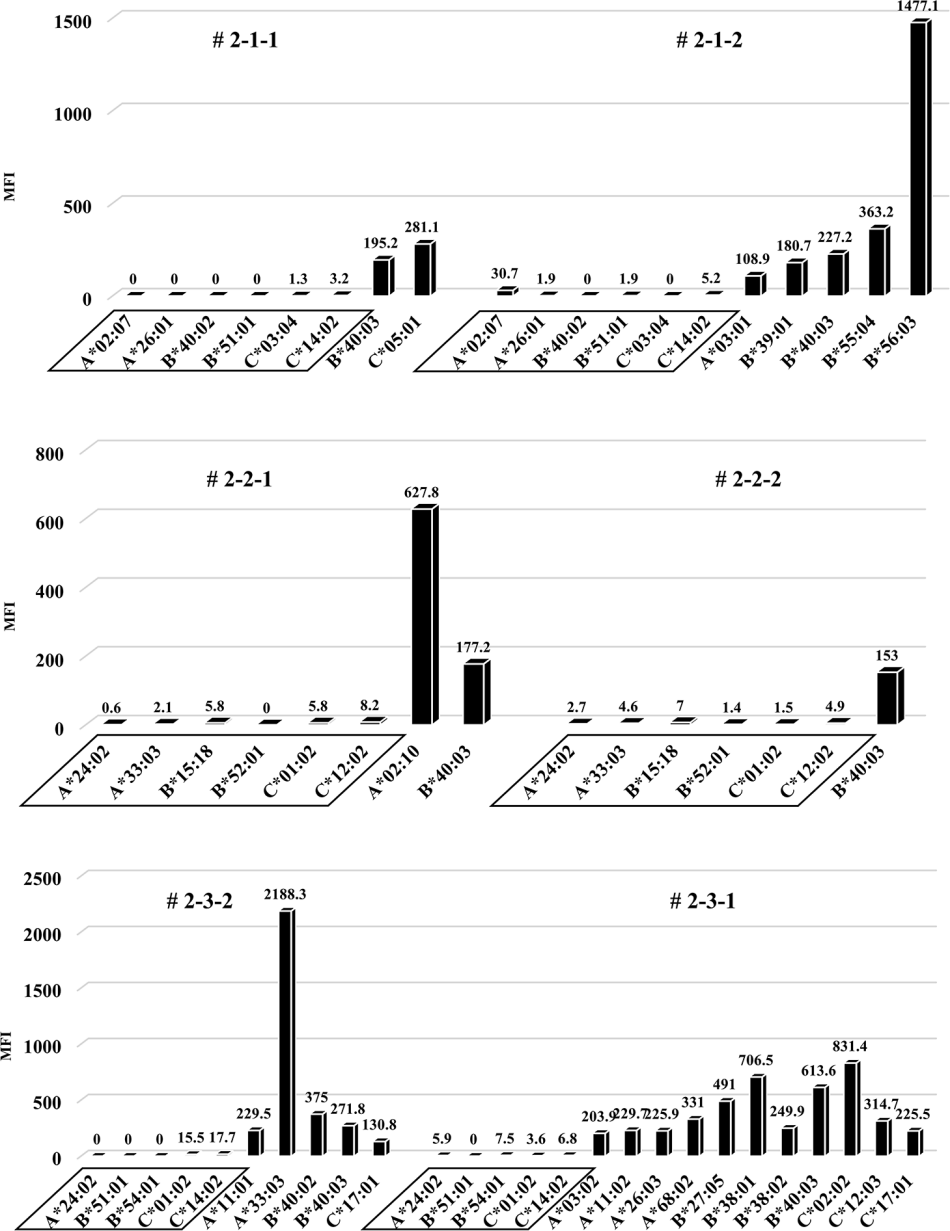
Anti-HLA Abs production in all humanized mice under the second protocol.

To induce better activation of antibody-producing B cells responding to HLA, we utilized the BAFF-BAFF receptor signaling system. This protocol was similar to the first but the feeder cells expressed both h-CD40L and h-BAFF. There were no major changes in the chimera status of the PBMCs from these humanized mice, compared with the first protocol (Fig 7). The quantification of human total-IgG and anti-HLA Abs were also similar to the first protocol. Further, there was no production of allospecific anti-HLA Abs, although the human total-IgG production level was sufficient (Figs 8 and 9). All the humanized mice from the second protocol had a small amount of the responder CD19^+^ B cells in their peripheral blood (Fig 7).

In FCM on day 14, only responder cells were detected in the peripheral blood of the humanized mice, but not stimulator cells. The cells derived from the responder is surrounded by square. This chimera status was the same as for the first protocol.

Humanized mouse sera were collected, and human total-IgG was measured as described in the first protocol, with the results showing the same tendency. Mouse # 2-2-2 was dead at day 34.

The results of anti-HLA Abs production in all humanized mice are shown. The stimulator HLA allele name (i.e., allospecific anti-HLA Abs) are enclosed in a square.

### No remarkable changes of results in human total-IgG, whole anti-HLA Ab production and allospecific anti-HLA Abs in the third protocol humanized mouse model

**Fig 10:**
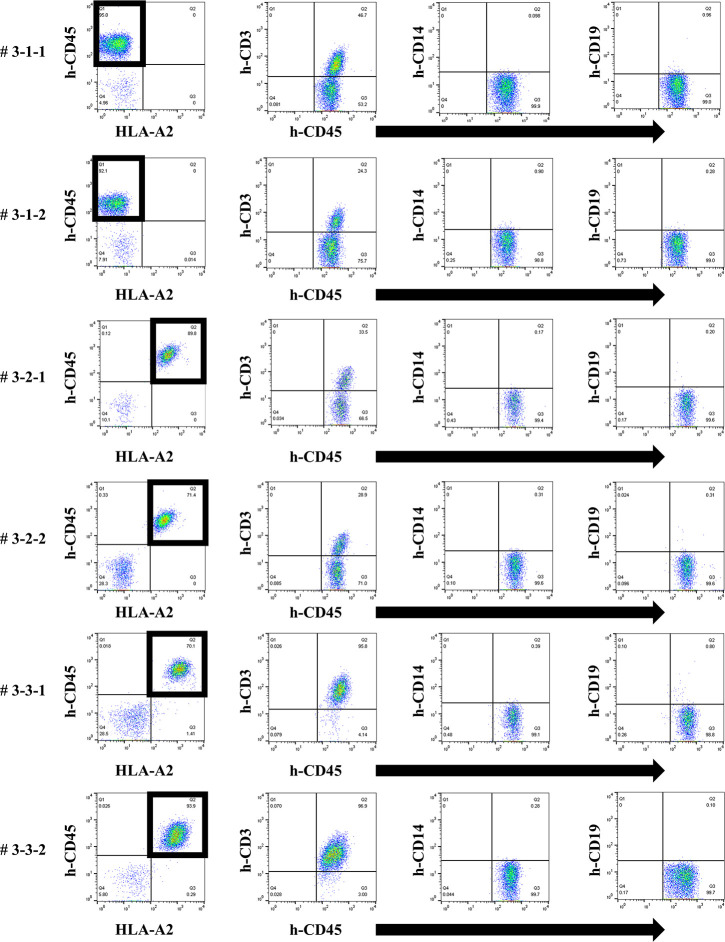
Chimeric status in all humanized mice under the third protocol.

**Fig 11:**
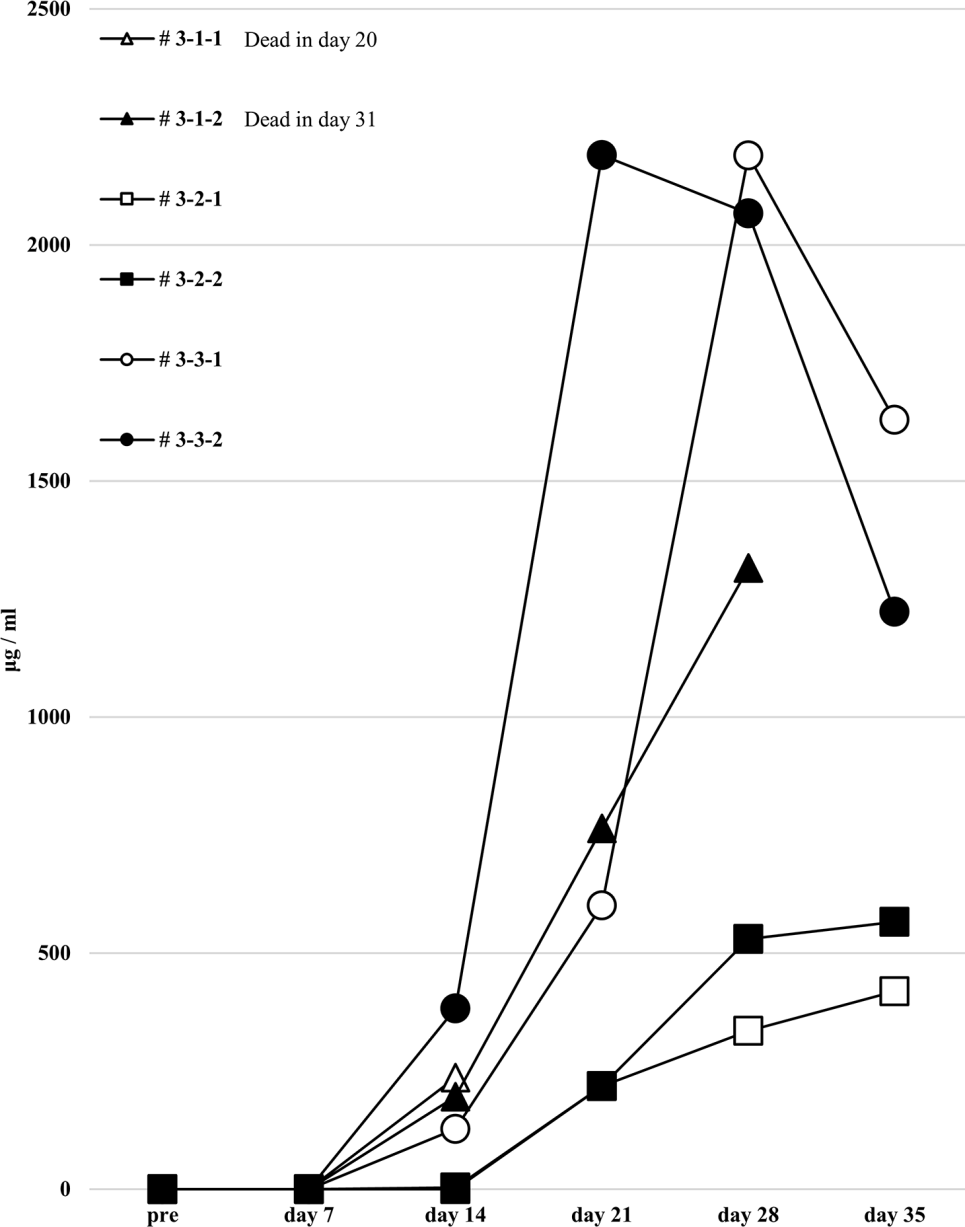
Human total-IgG production in all humanized mice under the third protocol.

**Fig 12:**
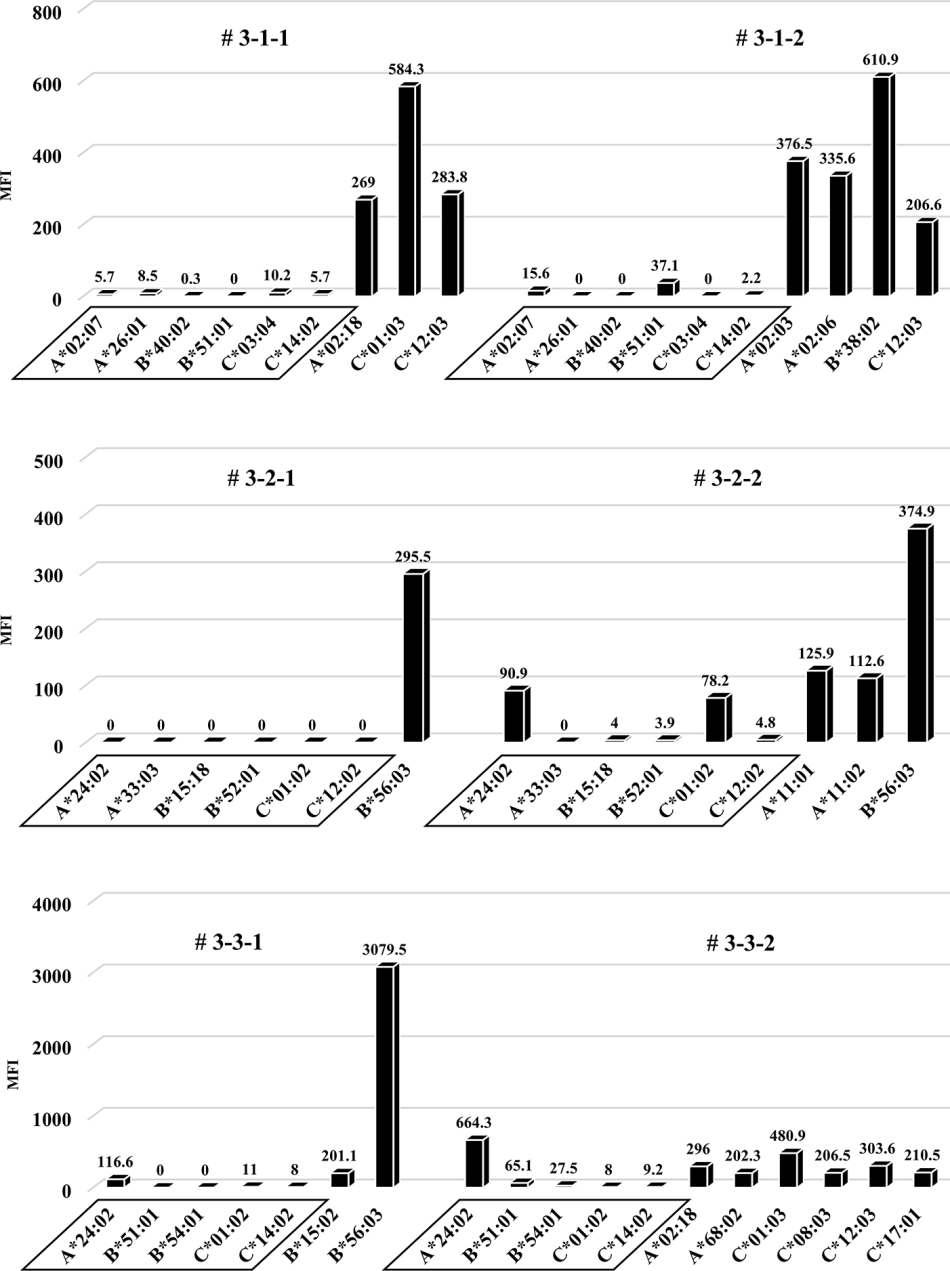
Anti-HLA Abs production in all humanized mice under the third protocol.

Considering that T-regs in the responder’s PBMCs may have interfered with the facilitation of the anti-HLA Ab production during culture, the same protocol as the first protocol was performed under conditions without T-regs, and the responder PBMCs were co-cultured with the feeder cells expressing h-CD40L. All results, including chimeric status, human total-IgG, and presence of anti-HLA Abs, were similar to the first protocol (Figs 10–12). Although a slight production of allospecific anti-HLA Abs were observed in mouse # 3-3-1 and # 3-3-2 (Fig 12), the proportion of responder B cells was not higher, nor were the human total-IgG titers remarkable.

In FCM on day 14, only the responder cells were detected in the peripheral blood of humanized mice under the third protocol. The cells derived from the responder is surrounded by square. This chimera status showed the same results as the first protocol.

Human total-IgG titers showed no remarkable changes in the third protocol. Mouse # 3-1-1 was dead at day 20 and # 3-1-2 was dead at day 31.

Quantification of anti-HLA Abs produced by all of the humanized mice under the third protocol are shown. The stimulator HLA allele name (i.e., allospecific anti-HLA Abs) are enclosed in a square. All humanized mice have produced various allelic non-specific anti-HLA Abs similar to the result in Fig 6. On the other hand, alloantigen specific anti-HLA Ab (A*24:02) was slightly produced in the mouse # 3-3-1 and # 3-3-2.

### Comparison of the titers in all protocols between allospecific and non-specific anti-HLA Abs

**Fig 13:**
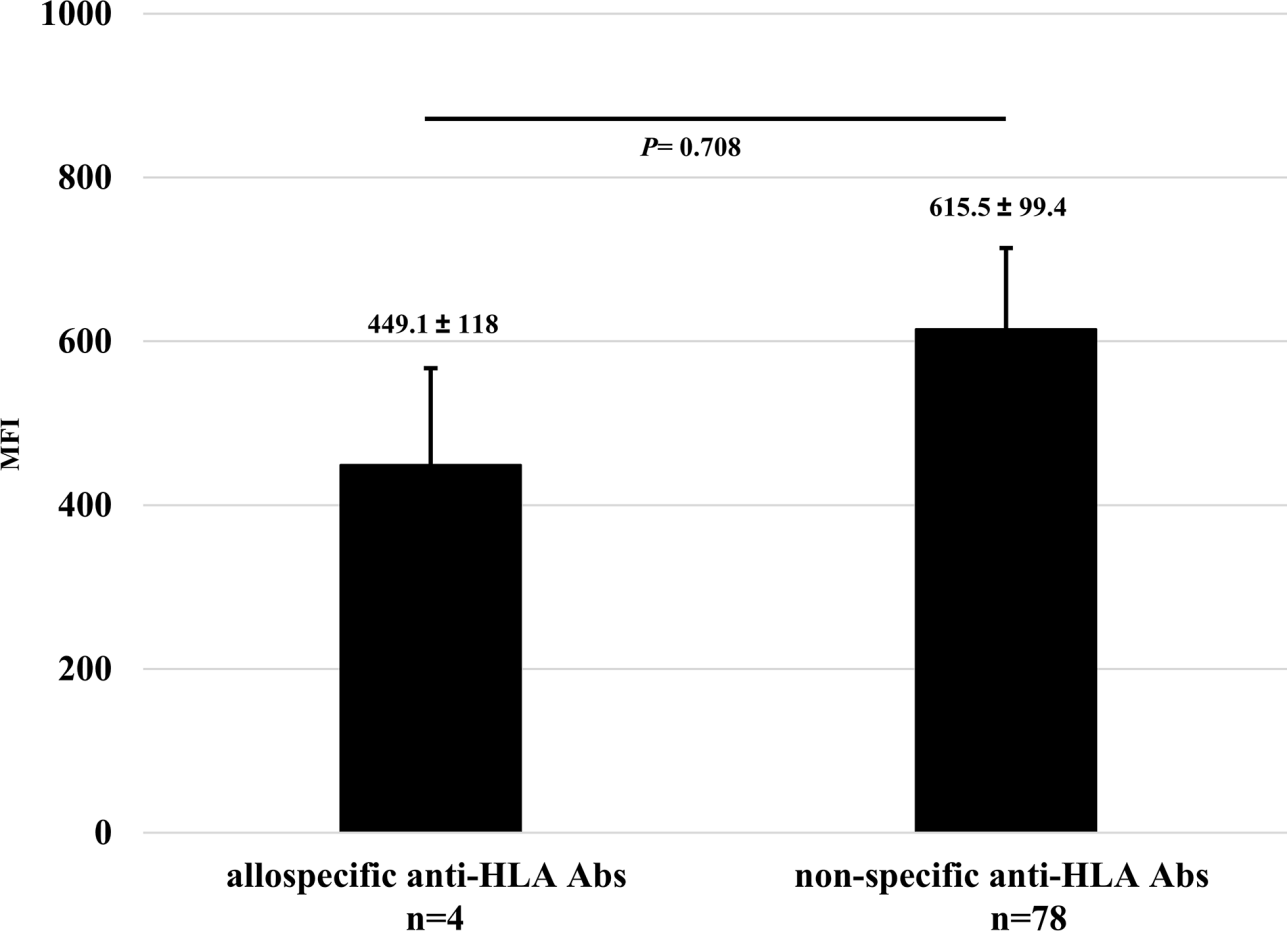
Comparison of the titers in all protocols between allospecific and non-specific anti-HLA Abs.

In all 9 experiments, the total number of anti-HLA Abs detected as positive was 82. Among them, the numbers of allospecific and non-specific anti-HLA Abs were 4 and 78, respectively. There was no statistically significant difference in mean MFIs between both the Abs (Fig 13).

Quantitative data was represented as the mean ± standard error. The mean MFIs of allospecific and non-specific anti-HLA Abs were 449.1±118 (range 116.6–664.3) and 615.5±99.4 (range 100–5567.5), respectively. Statistical analyses were performed using EZR.

## Discussion

Acute or chronic antibody-mediated rejection involving DSA is one of the most important issues to be overcome in clinical organ transplantation. However, the mechanisms and treatments of antibody-related rejection remain unresolved. The research of human immune responses and disease *in vivo* including anti-HLA Abs production are limited by many constraints. Animal models are needed that on the one hand accurately mirror the pathogenesis of disease, and on the other allow pre-clinical examination of human cell-based therapeutic approaches *in vivo*.

Moiser *et al*. reported in 1988 that injection of human peripheral blood leukocytes can result in the stable long-term reconstitution of a functional human immune system in mice with severe combined immunodeficiency (SCID) [14]. Since then, this humanized mouse model has been improved by using different immunodeficiency mouse strains. These humanized mouse models have been widely used for studies on cancer, infectious disease, transplantation or autoimmune disorders. Andrade *et al*. successfully constructed a reproducible systemic lupus erythematous (SLE)-humanized mouse model that produces human total-IgG and anti-double-stranded deoxyribonucleic acid Ab with PBMCs from SLE patients [15].

There are some standard approaches to introduce the human immune system into immunodeficient mice using PBMCs or hematopoietic stem cells (HSCs) in combination with implantation of autologous fragments of fetal thymus and liver [8, 10, 11]. The injection of human CD34^+^ HSC from bone marrow or cord blood into young mice is known as the Hu-RC-SCID model, that allows for the differentiation and development of the human immune system. Although the development of human T and B cells can be confirmed, insufficient maturation of these cells was observed [16, 17]. The reason for insufficient maturation is that the human cell differentiation promoting factor is not found in the mouse cells, and human cells are refractory to mouse cell differentiation promoting factor [17]. Although humanized mouse models using HSC have been widely used, a humanized mouse model having sufficient anti-HLA Ab-producing ability corresponding to allo-antigen from PBMCs has not still been established. One of the reasons is that sufficient Ab production in the mouse body may not have been achieved even if the HSC engraftment was excellent, perhaps because human T and B cell interaction in the mouse spleen germinal center was insufficient. Another reason is that class switching from IgM to IgG is less likely to occur, the antigen-specific Ab producing ability is extremely low, and the Ab production promoting factor in the mouse body is refractory to human B cells, as in the previously reported humanized mice. Also, it is considered that the differentiation potential of B cells is low [17]. Nevertheless, humanized mouse models using mature PBMCs but not HSCs are still frequently used in various studies. As shown in previous reports [16], mere administration of human PBMCs into immunodeficient mice did not result in anti-HLA Ab production. Thus, we hypothesize that human immune cells may not be in sufficient contact with each other in the mouse, and that allo-antigen stimulation may not be transmitted to the various responder immune cells including human T, B, and antigen-presenting cells in the mouse spleen. Given that *in vitro* mixed lymphocyte culture sufficiently transmits alloantigen stimulation to the antigen-presenting cells of responders, we speculated that transferring human responder matured and activated immunocompetent cells into the mouse after stimulating with alloantigen *in vitro* could produce allospecific anti-HLA Abs.

To this end, two types of human PBMCs were co-cultured *in vitro* with human B cell differentiation promoting factor before injecting into naïve NSG mice. Therefore, we considered that CD40-CD40L signaling is required as an Ab-producing B cell activating factor. Binding of CD40 on B cells by the CD40 ligand (CD154) on T cells promotes B cell proliferation, immunoglobulin production, isotype switching, and memory B cell generation [18]. The antigen-presenting cells (macrophage or dendritic cells) recognize an antigen, present the antigen to the T cells, and Ab production occurs by the interaction between the T cells and B cells (via CD40 and CD40L) [19–21]. We anticipated that antigen-specific Ab production might be enhanced by forcibly inducing the interaction via CD40 on the responder B cells by using feeder cells expressing h-CD40L. However, allospecific anti-HLA Abs were not sufficiently detected by stimulating of CD40L alone, as shown in Fig 6. There are reports that BAFF-BAFF receptor signaling system, in addition to CD40-CD40L signaling, is essential for mouse B cell differentiation and proliferation [22, 23]. As shown in Figs 8 and 9, the use of feeder cells expressing both h-CD40L and h-BAFF did not increase the titers of human total-IgG and anti-HLA Abs. Thus, although the induction of two typical signals that activate Ab-producing B cells resulted in sufficient anti-HLA Abs, production of allospecific anti-HLA Abs was still suppressed (Fig 9). As an opposing idea, we attempted removing regulatory T cells, which negatively control a function of Ab-secreting B cells. We suspected that the responder T-regs might only suppress the activation of antigen-specific B cells. Although an alternate B cell activation signal was added or the T-regs that negatively control Ab-producing cells removed, only non-specific anti-HLA Ab was produced, but antigen-specific anti-HLA Ab production remained suppressed (Fig 13).

These results suggest that these humanized mouse models have a mechanism of tolerance to allogeneic antigen-specific Abs production that is independent of the effects of h-BAFF-BAFF receptor signaling or T-reg regulation. This humanized mouse model is characterized by using mature human PBMCs from healthy volunteers rather than immature HSCs. Unlike the humanized mouse model administered with HSCs, one with PBMCs has the disadvantage of a shorter engraftment period that cannot be observed for a long time. However, in our experiment, since human PBMCs with a potential of producing some Abs are administered to mice under condition of Ab-producing B cell activation in vitro, the desired Ab production can be sufficiently identified within a relatively short period of 14–28 days. During the observation period, 5 out of 18 mice (# 1-1-2, # 1-2-1, # 2-2-2, # 3-1-1, # 3-1-2) died; we speculate this is due to the possibility that transferred cells recognized the mouse major histocompatibility complex and developed graft-versus-host disease. Presumably, the anti-HLA Abs were not directly affecting mouse tissue. Interestingly, although we did not obtain alloantigen-specific anti-HLA Abs, we produced sufficient amounts of non-specific HLA Abs in the humanized mice. We supposed that this may be due to induction of alloantigen-specific tolerance during the *in vitro* culture with h-CD40L expressing mouse feeder cells, or the B and T cells that respond to alloantigen were exhausted and did not reach high enough levels to produce allo-specific anti-HLA Ab. Also, we guessed that B and T cell clones that respond to alloantigen in the *in vitro* mixed lymphocyte reaction may have been removed or induced anergy during the culture, such that development of a humanized mouse model in which allo-specific anti-HLA Abs were not produced. In combination 3, allospecific anti-HLA Abs (A*24:02) were produced to some extent. We guessed the responder PBMCs may have been sensitized to HLA-A*24:02 originally. However, when the anti-HLA Ab titers in the sera of the three responders in all the experiments were measured, none of the Abs against the HLA antigen of the corresponding stimulators was detected. Therefore, the reason for the slight detection of this positive response to (A*24:02) is unknown.

We had initially tried to establish a humanized mouse model that produces allo-specific anti-HLA Abs. Conversely, a tolerant humanized model that produces non-specific but not allospecific anti-HLA Abs was established. From these results, this anti-HLA Ab-producing humanized mouse model might be useful as a novel and profound model that can induce only antigen-specific B cells to tolerance.

## Conclusions

We attempted to develop an allospecific anti-HLA Abs-producing humanized mice conditioned with human PBMCs. As a result of culturing feeder cells expressing both h-CD40L and h-BAFF for the purpose of activating Ab-producing B cells, sufficient non-specific anti-HLA Abs production was obtained, whereas allospecific anti-HLA Ab production was unexpectedly suppressed. The availability and presence of a mechanism to negatively control the production of antigen-specific Abs in the *in vitro* culture system might make this anti-HLA Ab-producing humanized mouse a useful and profound model for the induction of tolerance by antigen-specific B cells. However, further investigations to elucidate the allogenic-specific tolerance mechanism in the modified humanized mouse model are needed.

## Supporting information

S1 Fig Expression of h-BAFF in FCM. After transfection into the h-CD40L expressing NIH 3T3 fibroblasts, the rate of h-BAFF expression was over 90%.
(TIF)

S2 Fig The ratio before and after T-regs removal in FCM. The initial proportion of T-regs in PBMC was 16.2% (A) and was decreased to 1.2% after removal of T-regs (B) in FCM.
(TIF)

S3 Fig Flowchart of the established humanized mouse model producing anti-HLA Abs. The flow of the protocols to establish humanized mice producing anti-HLA Abs is shown.
(TIF)
