# Supplementary figures and images for: Development of a humanized mouse model to analyze antibodies specific for human leukocyte antigen (HLA)
Source: PLoS One. 2021 Feb 5;16(2):e0236614. doi: 10.1371/journal.pone.0236614 (PMC7864411; doi:10.1371/journal.pone.0236614)

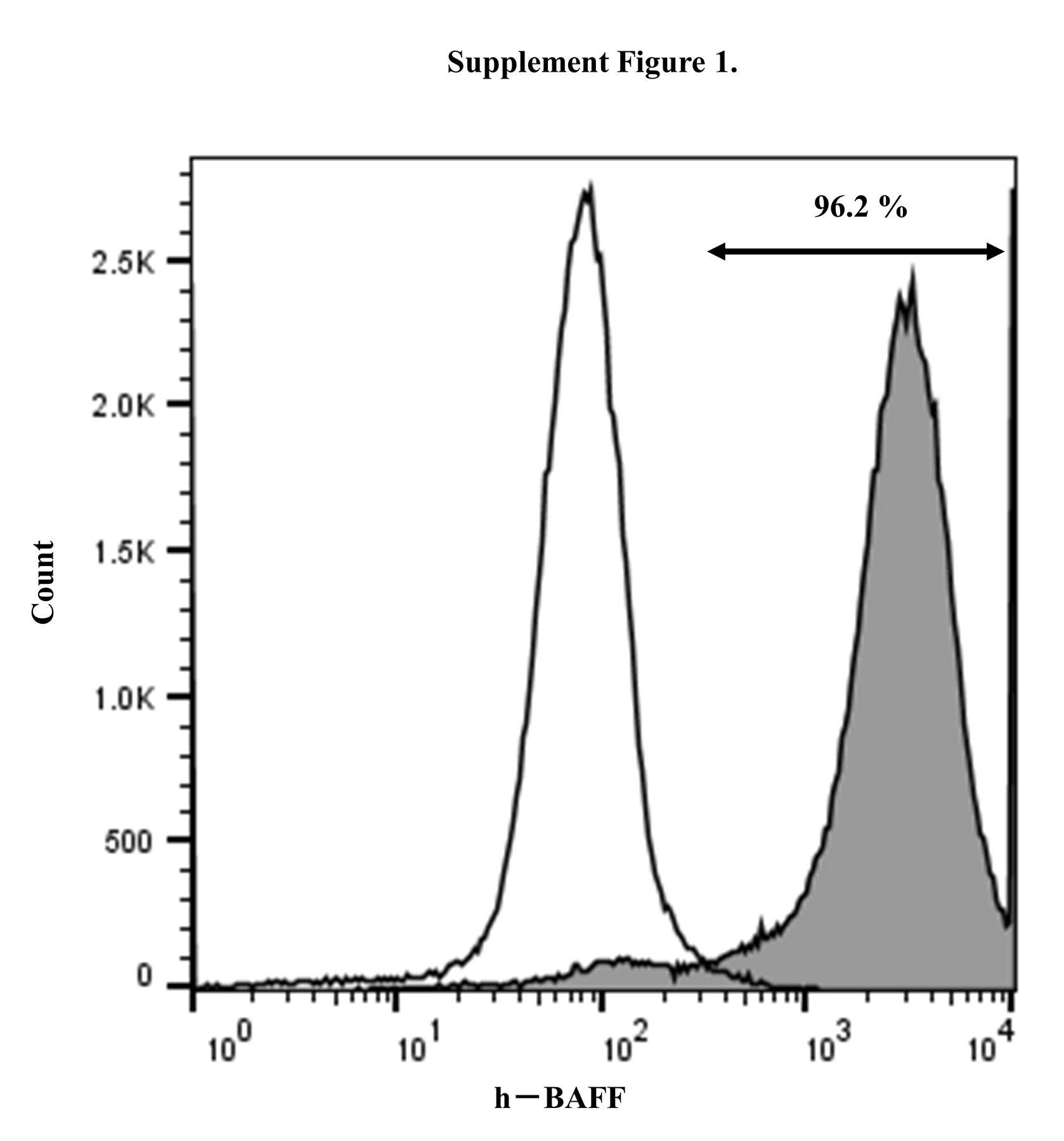

Supplement: S1 Fig — After transfection into the h-CD40L expressing NIH 3T3 fibroblasts, the rate of h-BAFF expression was over 90%. (TIF) [file pone.0236614.s001.tif]

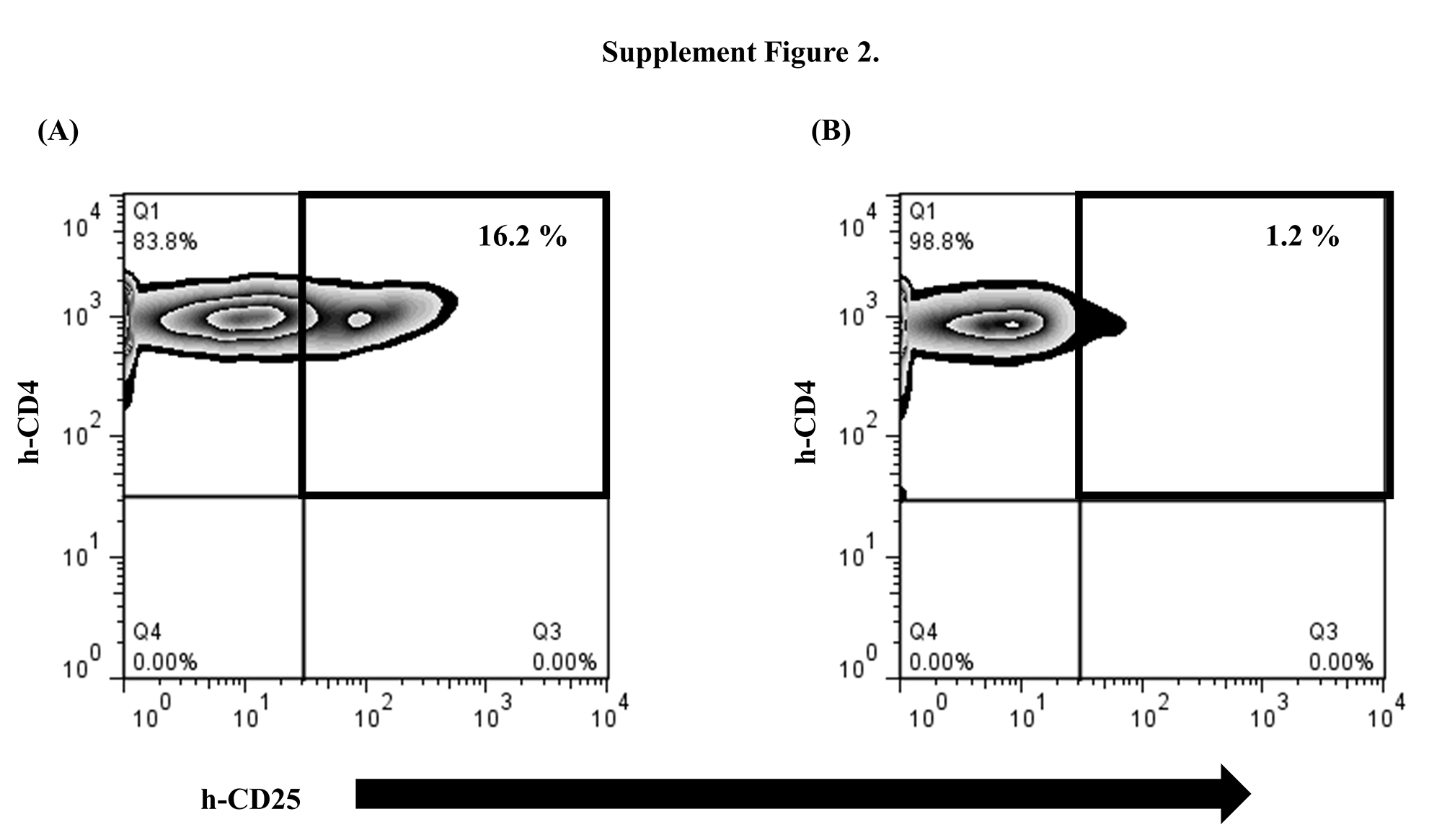

Supplement: S2 Fig — The initial proportion of T-regs in PBMC was 16.2% (A) and was decreased to 1.2% after removal of T-regs (B) in FCM. (TIF) [file pone.0236614.s002.tif]

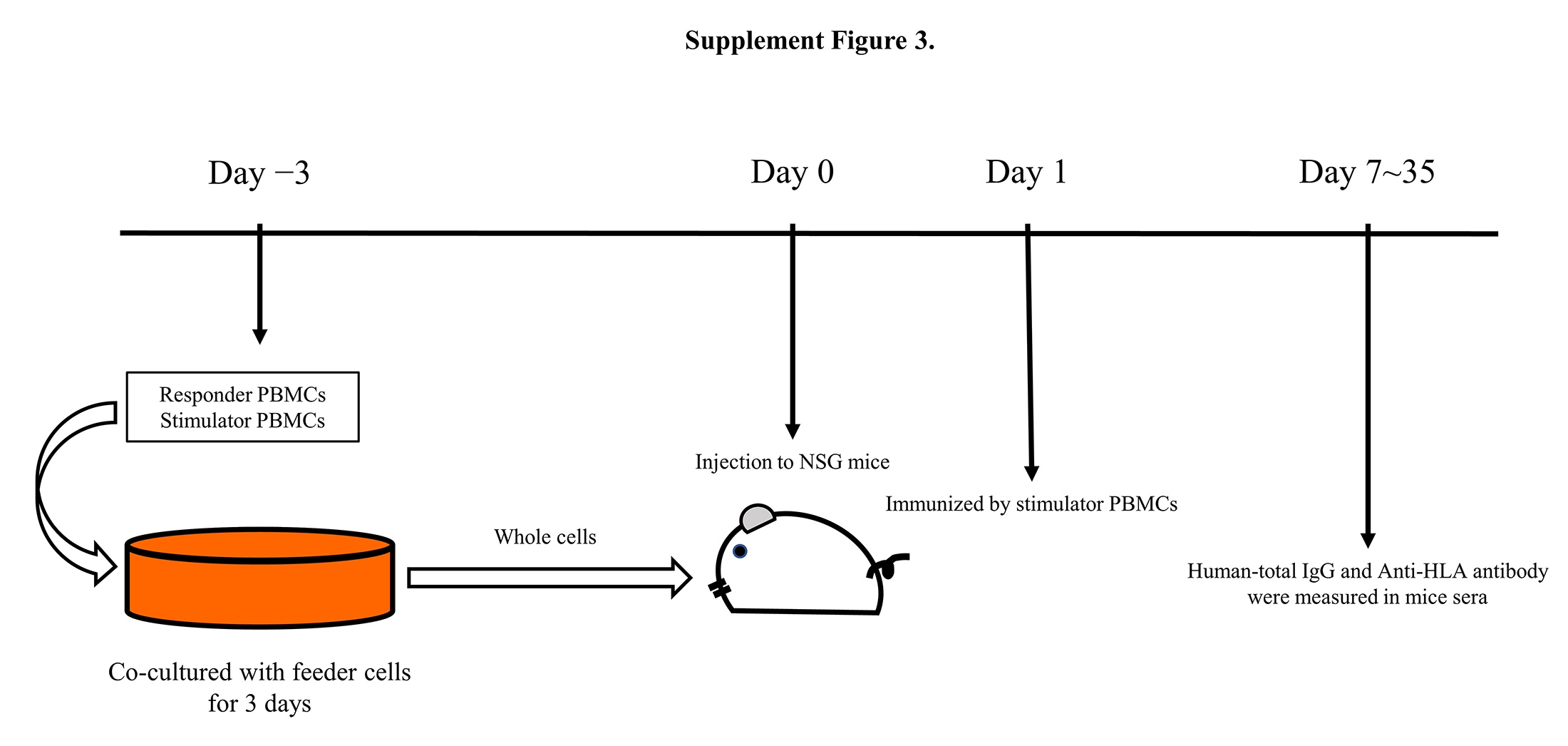

Supplement: S3 Fig — The flow of the protocols to establish humanized mice producing anti-HLA Abs is shown. (TIF) [file pone.0236614.s003.tif]
